# Granular Parakeratosis With Spontaneous Resolution: A Case Report

**DOI:** 10.7759/cureus.24085

**Published:** 2022-04-12

**Authors:** Ghada Alhayaza, Meshal Alessa, Ohoud Alsaedi, Ahmed Alhumidi, Maram Alzain

**Affiliations:** 1 Dermatology and Dermatologic Surgery, Prince Sultan Military Medical City, Riyadh, SAU; 2 College of Medicine, Dar Al Uloom University, Riyadh, SAU; 3 Department of Pathology, College of Medicine, King Saud University, Riyadh, SAU

**Keywords:** spontaneous resolution, self-limiting, -dermatopathology, dermatology, granular parakeratosis

## Abstract

Granular parakeratosis (GP) is a rare, idiopathic, and self-limiting cutaneous disorder. It clinically presents as erythematous to brown hyperkeratotic or scaly papules that can coalesce to form plaques. If GP is suspected clinically, histopathological confirmation is adequate for diagnosis. Several treatment modalities were tried with varying success, but none was consistently efficacious.

Given the rarity of GP and the variety in its clinical presentation and management, we report a case of a self-resolving infra-abdominal GP. Our patient is a 47-year-old female who presented with a one-week history of asymptomatic, multiple, linear, horizontal, brown, hyperpigmented scaly papules in the infra-abdominal fold. She had a three-year history of applying almond oil and Sudocrem Antiseptic Healing Cream®. Histopathology showed the retention of basophilic keratohyalin granules within the area of parakeratosis in the stratum corneum, which is consistent with GP. She was discharged on emollients, and on follow-up one month later, her lesions completely resolved.

In conclusion, GP is a rare cutaneous disorder characterized by hyperkeratotic plaques or papules typically on intertriginous areas. The natural history of the disease may vary from spontaneous resolution to a waxing and waning condition. In addition, given how uncommon the disease is and its variable etiologies and course, definite management is yet to be established and a standardized treatment recommendation is lacking.

## Introduction

Granular parakeratosis (GP) is a rare, idiopathic, and self-limited cutaneous disorder. It was first described by Northcutt et al. in 1991, as an axillary eruption with a unique histopathological finding and termed “axillary granular parakeratosis.” Since then, it has been described in other intertriginous and non-intertriginous body parts, eliminating the word “axillary” [1]. It clinically presents as erythematous to brown hyperkeratotic or scaly papules that can coalesce to form plaques [1-2]. Multiple theories were raised to understand the etiology of GP, but none have been confirmed as solely the cause [1,3]. If GP is suspected clinically, histopathological confirmation is adequate for diagnosis. Histopathologic findings include a thickened stratum corneum with keratohyalin granules [2]. Several treatment modalities have been attempted with varying success, but there is no consistently efficacious therapy [1]. Given the rarity of this disorder, we present in this report a case of infra-abdominal-fold granular parakeratosis in a middle-aged female with spontaneous resolution in a few weeks.

## Case presentation

**Figure 1:**
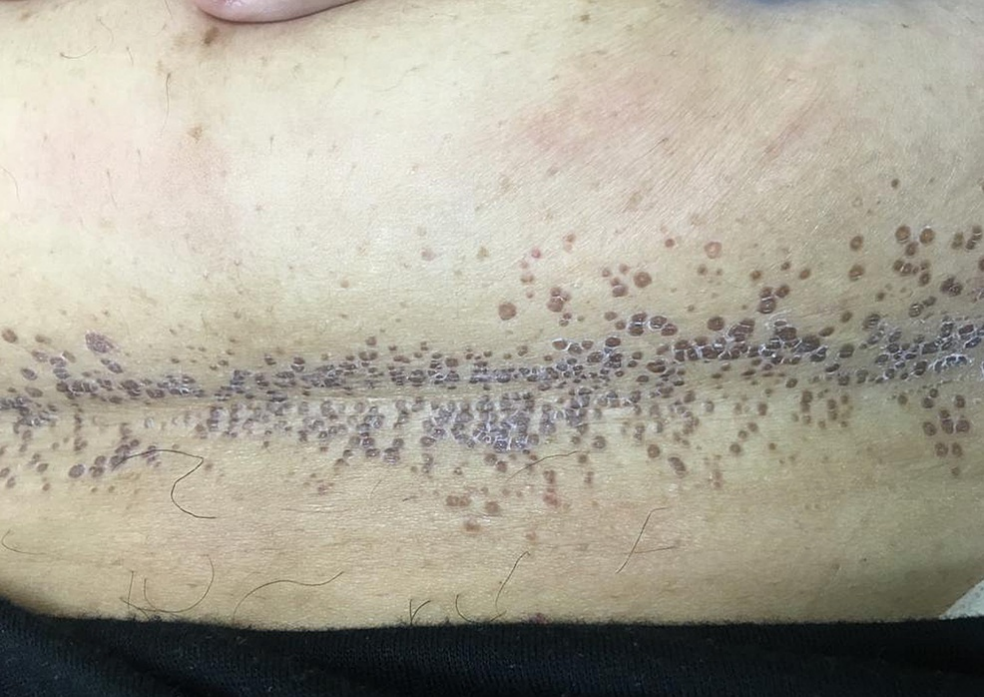
Infra-abdominal fold of the patient

**Figure 2:**
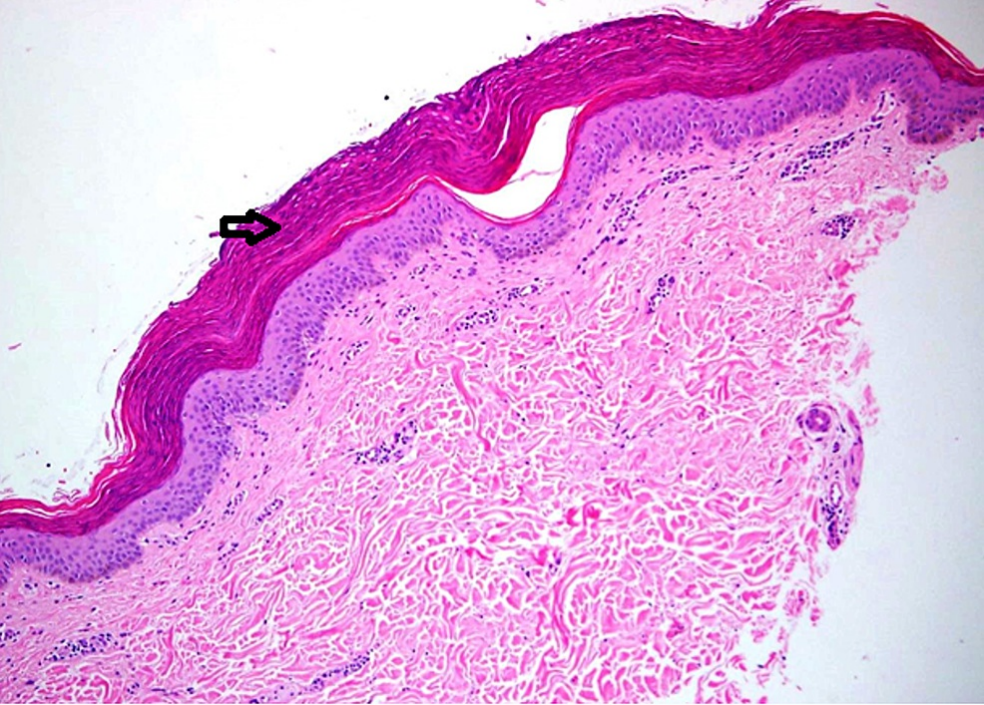
Photomicrograph of the skin punch biopsy revealing thickened keratin layer (arrow) Hematoxylin and eosin (H/E) stain Original magnification x40

**Figure 3:**
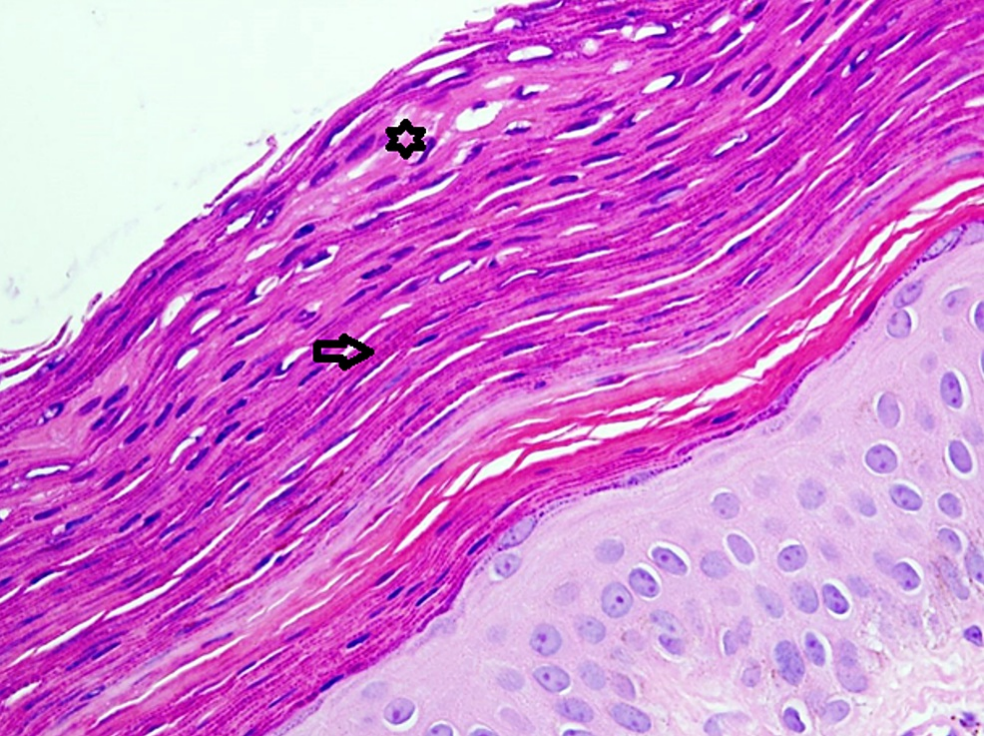
High-power view of the skin biopsy showing parakeratosis (star), with visible, retained basophilic keratohyalin granules (arrow) Hematoxylin and eosin (H/E) stain Original magnification x400

A 47-year-old woman presented to our dermatology clinic with asymptomatic skin lesions on the abdomen for almost a week. She was obese with a body mass index (BMI) of 39. Her medical and surgical history was unremarkable. She mentioned applying Sudocrem Antiseptic Healing Cream® in combination with almond oil on her body folds for almost three years prior to the appearance of the lesions. Upon examination, there were multiple, brown, hyperpigmented, scaly, flat-topped papules in a linear horizontal configuration in the infra-abdominal fold (Figure 1). A three-millimeter skin punch biopsy was taken from a lesion for histopathology. On hematoxylin and eosin (H&E) staining, there was a thickened keratin layer with parakeratosis (Figure 2). Further, high-power microscopy exhibited the retention of basophilic keratohyalin granules within the area of parakeratosis in the stratum corneum (Figure 3). In addition, the Periodic acid-Schiff (PAS) stain was negative for fungal microorganisms.

The patient was given emollients with no intervention. Upon follow-up a month later, the skin lesions had completely spontaneously resolved almost 20 days after the initial appearance. Three years later, there was no recurrence.

## Discussion

Multiple theories exist on the possible etiology of GP [1]. The initial theory by Northcutt et al. was that it is a contact reaction to topical products, leading to an interruption in the breakdown of profilaggrin to filaggrin in the course of epidermal differentiation [4]. Similarly, our patient applied Sudocrem Antiseptic Healing Cream®, which contains zinc oxide, lanolin, benzyl alcohol, benzyl benzoate, benzyl cinnamate, sodium benzoate (benzoic acid), linalyl acetate, lavender, beeswax, butylated hydroxyanisole, sorbitan sesquioleate, liquid paraffin, paraffin wax, microcrystalline wax, and citric acid. However, cases were also reported in the absence of the use of topical products [2]. Other theories include occluded skin folds and obesity as an etiology [5]. In addition, our patient was obese, which might highlight it as a precipitating factor. Another theory is that GP is caused by an acquired disorder of cornification with unknown etiology, unrelated to contact [6-7]. This theory is backed by the response of GP to isotretinoin therapy when the precipitating trigger is not identified [7]. Lastly, even though infectious causes were not adequately attributed to the etiology, some cases responded to oral antibiotics [8]. For the previous reasons, GP can be considered a reaction pattern rather than a distinct disease.

Clinically, GP can be confused with other disorders, especially those affecting the folds like Hailey-Hailey disease, erythrasma, and retention hyperkeratosis among others. In addition, it can be present in association with conditions like tinea corporis but not any systemic diseases [3]. Patients with GP typically present with either asymptomatic or pruritic, reddish to brown, scaly or hyperkeratotic papules and plaques. The skin lesions can arise over several days to months [5].

With high clinical suspicion, a skin biopsy is required for histopathological diagnosis [2]. In addition, a skin biopsy can delineate if GP is present alone or associated with a treatable condition like tinea corporis [9]. On H&E staining, the highlight of the histopathologic features is seen as a thickened stratum corneum with an intact stratum granulosum. These features include hyperkeratosis, parakeratosis, and retention of keratohyalin granules, which give this layer an intense blue appearance [1]. Furthermore, electron microscopy can show a granular layer with round or stellate keratohyalin granules and retained keratohyalin granules within the corneocytes in the corneal layer [9].

Managing patients with GP can be challenging since responses to treatments have been inconsistent [3]. A wide range of treatments has been used, including removal of the possibly offending agent, behavioral therapy, cryotherapy and other ablative modalities, and botulinum toxin injections even with the absence of a history of hyperhidrosis [4-5,10]. Also, oral and topical retinoids, antibiotics, antifungals, topical vitamin D analogs, topical corticosteroids, ammonium lactate, and topical calcineurin inhibitors have been tried with varying results [4-6,10]. In addition, spontaneous resolution has been reported and usually takes from one month to one year [6]. Similarly, our patient had spontaneous resolution 20 days later with no intervention even though she did not completely stop applying Sudocrem Antiseptic Healing Cream® and almond oil.

## Conclusions

In conclusion, GP is a rare cutaneous disorder characterized by hyperkeratotic plaques or papules typically on intertriginous areas. The natural history of the disease may vary from spontaneous resolution to a waxing and waning condition. In our case, the patient had a spontaneous resolution in less than a month. Her obesity and application of topical preparations were possible contributing factors to the condition. In addition, due to the rarity of the disease and the variable etiologies and course, definite management is yet to be established and a standardized treatment recommendation is lacking.
